# Deterministic mathematical models of the cAMP pathway in *Saccharomyces cerevisiae*

**DOI:** 10.1186/1752-0509-3-70

**Published:** 2009-07-16

**Authors:** Thomas Williamson, Jean-Marc Schwartz, Douglas B Kell, Lubomira Stateva

**Affiliations:** 1 Faculty of Life Sciences, The University of Manchester, Manchester, M13 9PT, UK; 2 School of Chemistry, The University of Manchester, Oxford Rd, Manchester M1 9PL, UK; 3 Manchester Centre for Integrative Systems Biology, Manchester Interdisciplinary Biocentre, The University of Manchester, 131 Princess St, Manchester M1 7DN, UK

## Abstract

**Background:**

Cyclic adenosine monophosphate (cAMP) has a key signaling role in all eukaryotic organisms. In *Saccharomyces cerevisiae*, it is the second messenger in the Ras/PKA pathway which regulates nutrient sensing, stress responses, growth, cell cycle progression, morphogenesis, and cell wall biosynthesis. A stochastic model of the pathway has been reported.

**Results:**

We have created deterministic mathematical models of the PKA module of the pathway, as well as the complete cAMP pathway. First, a simplified conceptual model was created which reproduced the dynamics of changes in cAMP levels in response to glucose addition in wild-type as well as cAMP phosphodiesterase deletion mutants. This model was used to investigate the role of the regulatory Krh proteins that had not been included previously. The Krh-containing conceptual model reproduced very well the experimental evidence supporting the role of Krh as a direct inhibitor of PKA. These results were used to develop the Complete cAMP Model. Upon simulation it illustrated several important features of the yeast cAMP pathway: Pde1p is more important than is Pde2p for controlling the cAMP levels following glucose pulses; the proportion of active PKA is not directly proportional to the cAMP level, allowing PKA to exert negative feedback; negative feedback mechanisms include activating Pde1p and deactivating Ras2 via phosphorylation of Cdc25. The Complete cAMP model is easier to simulate, and although significantly simpler than the existing stochastic one, it recreates cAMP levels and patterns of changes in cAMP levels observed experimentally *in vivo *in response to glucose addition in wild-type as well as representative mutant strains such as *pde1Δ, pde2Δ*, *cyr1Δ*, and others. The complete model is made available in SBML format.

**Conclusion:**

We suggest that the lower number of reactions and parameters makes these models suitable for integrating them with models of metabolism or of the cell cycle in *S. cerevisiae*. Similar models could be also useful for studies in the human pathogen *Candida albicans *as well as other less well-characterized fungal species.

## Background

**Figure 1:**
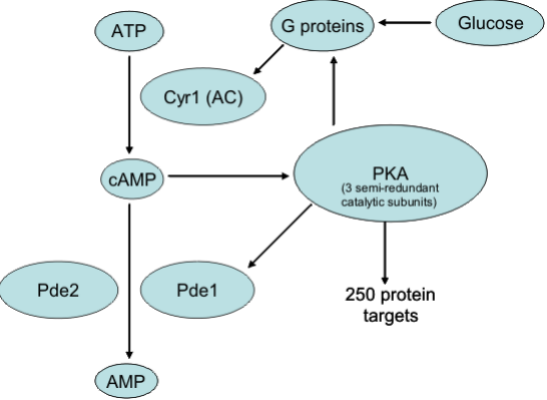
**Schematic representation of elements of the cAMP pathway in *S. cerevisiae***.

Cyclic adenosine monophosphate (cAMP) is an important signalling and regulatory molecule. In eukaryotes cAMP activates Protein Kinase A (PKA), the target kinase of the cAMP-mediated signal transduction pathway. In the widely used model baker's yeast *Saccharomyces cerevisiae*, this pathway regulates a variety of cellular processes, including metabolism [1], response to stress [2,3] and progression through the cell cycle [4,5]. The pathway is modulated by external nutrients, most notably glucose [6]. The transition to growth on glucose in yeast is orchestrated by a tightly regulated pattern of changes in cAMP levels as a result of series of interactions involving the components of the cAMP/PKA pathway (Figure 1). Cyclic AMP is synthesized by adenylate cyclase (Cyr1p), which in turn is regulated by Gpa2p [7] and Ras2p [8], both of which are G proteins. Gpa2p is activated by the G-protein-coupled receptor Gpr1p, which in turn is activated by glucose [9]. Gpa2p is deactivated by the regulator of G protein signalling protein (RGS) Rgs2p, as well as its own intrinsic GTPase activity [10]. Ras2p is activated by the guanine-nucleotide-exchange factor (GEF) Cdc25p [11] and Sdc25p [12], and deactivated by the GTPase activating proteins (GAPs) Ira1p and Ira2p [13]. The level of intracellular GTP is believed to influence the level of GTP-bound Ras2p [14], and the GTP level increases following a pulse of glucose [13], although the mechanism behind this increase is not fully understood.

The cAMP level is modulated by two phosphodiesterases: Pde2p has higher affinity for cAMP (around 1 × 10^-3 ^mM) [15] compared to Pde1p which has a lower affinity for cAMP in crude extracts (around 0.1 mM) [16,17]. Yeast cells previously starved for glucose exhibit a characteristic "spike" of cAMP following addition of glucose to the growth media. In wild-type cells, this spike reaches a peak at around 60 seconds before reaching a steady level after around 120 seconds

In the yeast cell, the only known function of cAMP is to activate protein kinase A (PKA). A molecule of PKA consists of two regulatory (R) and two catalytic (C) subunits. Under low cAMP concentrations, the R and C subunits are bound together to form a catalytically inactive heterotetramer. The complex is activated when two molecules of cAMP bind to each R subunit, causing their dissociation from the catalytic subunits. Following dissociation, the free C subunits can phosphorylate their targets. In yeast, the R subunit is encoded by *BCY1*, while the C subunits are encoded by the partially redundant genes *TPK1*, *TPK2 *and *TPK3*. Recently specific as well as common phosphorylation targets of the Tpk isoforms have been identified [18].

PKA exerts feedback on the system in several ways. First, it has been shown that the low affinity cAMP phosphodiesterase Pde1p is phosphorylated following a glucose pulse and Pde1p can be phosphorylated by bovine PKA [19]. Phosphorylation of Pde1p leads to increased phosphodiesterase activity, which plays a part in reducing the cAMP level following a glucose induced spike. Secondly, PKA can phosphorylate Cdc25p, leading to its dissociation from Ras2p [20]. This results in a decrease in adenylate cyclase activity. Finally, PKA may be able to regulate itself, as it has been demonstrated that Tpk1p is phosphorylated following a glucose pulse [21].

The roles of certain components of the cAMP pathway are still disputed. One of them is that of the Kelch Repeat Homologue proteins Krh1 and Krh2, also known as Gpb1 and Gpb2, as they are believed to function as beta subunits of Gpa2p. According to Harashima and Heitman [22] the Krh proteins stabilize the Ira proteins, the GTPases of the Ras proteins. Deletion of the Krh proteins leads to a loss of the Ira proteins, and therefore cAMP signalling is increased. However, there is evidence that shows that the Krh proteins enhance the association between the regulatory and catalytic subunits of PKA, and this enhancement is removed when the Krh proteins form a complex with activated Gpa2 [7,23]. Further evidence for the role of the Krh proteins comes from studies of adenylate cyclase (*cyr1Δ*) mutants [7]. Yeast *cyr1Δ pde2Δ *mutants can survive on YPD supplemented with 5 mM cAMP. However, the quadruple *cyr1Δpde2Δkrh1Δkrh2Δ *mutants survive in the presence of 1 mM cAMP, suggesting that the Krh proteins directly inhibit PKA activity, as PKA activity is necessary for yeast survival. In addition a *cyr1Δpde2ΔGPA2*^*Q*300*L *^mutant (with Gpa2 locked in its constitutively active GTP bound state) requires 1 mM cAMP for survival. This gives further support to the theory that Krh is recruited to active Gpa2.

The reductionist approach [24] has taught us much about individual elements of the cAMP pathway; however a quantitative and integrated mathematical representation is needed to fully understand its dynamics. Models of two broad categories can be used for this purpose: deterministic and stochastic [25-27]. Deterministic models which usually consist of a series of ordinary differential equations (ODEs) to describe the system in respect to time, have been used to study yeast systems such as glycolysis [28], the pheromone pathway [29-31] and the cell cycle [32]. Stochastic models on the other hand are used when intrinsic noise is important to the system, such as when low species numbers are involved [33]. However, stochastic models can be computationally expensive to simulate [34].

A stochastic model has been developed to examine the effects of altering the intracellular GTP levels on the Ras/cAMP/PKA pathway [14]. However, in yeast the components of the cAMP pathway are present in high numbers (proteins in thousands, nucleotides in millions) making a deterministic model more appropriate. Moreover, this stochastic model did not include the Krh proteins. In this study we present a deterministic mathematical model of the yeast Ras/PKA/cAMP pathway, with components such as the Krh proteins that have not been included before. Our model has been fitted to experimental data. It is much easier to simulate than is the previously reported stochastic model, yet it can faithfully replicate intracellular species concentrations observed at steady state, and following a perturbation of the system with glucose.

## Methods

ODE models of biochemical systems consist of variables and parameters. The variables represent species concentrations, whereas the parameters include rate coefficients, kinetic parameters, etc. If we represent the variables (*x*_*i*_) as a vector *X*:

(1)

and the parameters (*k*_*i*_) as a vector *θ*:

(2)

then an ODE model can be represented with the following equation:

(3)

The models generated in this study are summarised in Table 1. The reaction formulae which form the basis of the models were entered into Gepasi [35] and/or Copasi [25], and these programs were used for earlier inspection of the models. The models were later exported in Systems Biology Markup Language (SBML) format [36], which allowed the models to be exchanged between programs. SBToolbox in Matlab [37] was used for parameter estimation, parameter sensitivity analysis and model simulations.

**Table 1:** Summary of the models generated in this study.

**Model name**	**No. of parameters**	**No. of variables**	**Description**
PKA Model A	5	9	Deterministic model of the PKA module based on Cazzaniga *et al *[14]

PKA Model B	5	9	PKA Model A with optimized parameter values

PKA Model C	2	3	Simplified PKA module with mass action kinetics

PKA Model D	2	4	Simplified PKA module with Michaelis-Menten kinetics

Simplified cAMP Model A	16	5	Conceptual model of the entire cAMP pathway

Simplified cAMP Model B	18	6	Simplified Model A modified to include Krh proteins

Complete cAMP Model	27	15	Complete model of the cAMP pathway with estimated parameters

Steady state parameter sensitivity analysis was carried out according to the following equation:

(4)

where *S*_*ij *_is the sensitivity of species *i *in relation to parameter *j*, *Xss*_*i *_is the steady state level of species *i*, *p*_*j *_is the value of parameter *j*, and *Δp*_*j *_is a perturbation of parameter *j *(equal to 1% of the parameter value).

Cyclic AMP time course data were taken from the literature [19,38]. As cAMP levels are often reported in terms of nanomoles per gram of wet weight (or equivalent) it was necessary to convert them to nanomolar using the following formula:

(5)

where *C(nM) *is the nanomolar concentration of cAMP, *C*(*nmolesgww*^-1^) is the cAMP concentration in nanomoles per gram of wet weight reported in the literature, *Cw *is the conversion factor from grams wet weight to grams dry weight (0.15) and *Vc *is the volume of 1 × 10^7 ^cells in litres (2.68 × 10^-6^, there are approximately 1 × 10^7 ^cells in 1 gram of dry weight).

We recognise that ODE models of this type assume that all cells are identical, which may well not be the case [39].

### Parameter estimation

The values of system parameters which were not experimentally derived, were fitted to experimental cAMP time course data using simulated annealing [40,41], an estimation method that is very efficient in finding a close approximation of the global minimum of an optimization problem. It is based on a probabilistic search, in which every iteration of the algorithm replaces the current solution by a random nearby solution, using a probability distribution that tends to move the solution towards the global minimum. The simulated annealing algorithms found in SBToolbox in Matlab [37] with the SBToolbox function SBparameterEstimation were used for parameter estimation in the current study.

## Results

### The Protein Kinase A module

The only known biochemical role of cAMP is to activate PKA. This process has a complicated reaction scheme, which is challenging to model. A general guiding principle when building models is to make the model as simple as possible, while capturing realistic behaviour [42]. The expected behaviour of any PKA model must be consistent with the currently available experimental evidence. Firstly, a degree of PKA activity is required for cell viability [43]. If no cAMP is present, the cell is nonviable [44]; therefore all catalytic subunits must be contained within the inactive tetramer in the absence of cAMP. The level of free catalytic subunits must be sensitive to the level of cAMP. The cAMP level can range from 0.015 mM in glucose starved cells, to approximately 0.05 mM (a peak of cAMP induced by a glucose pulse) [38].

The stochastic PKA module reported by Cazzaniga *et al *[14] makes several assumptions. The binding constants for the association of a cAMP molecule with the PKA tetramer are the same for all cAMP bound states of PKA, as well as the dissociation constants. The underlying assumption is that cAMP binds to PKA in a non-cooperative manner, i.e. the binding of a molecule of cAMP to PKA does not affect the binding/dissociation of further cAMP molecules. In addition, the dissociation of the cAMP-bound PKA holoenzyme, and the subsequent dissociation of cAMP from the free R subunit is considered to be very fast, as is the reassociation of the PKA holoenzyme. We have adopted the same assumptions for our deterministic model.

The stochastic PKA module found in [14] can be converted into a series of deterministic ODEs to give PKA Model A (Table 1). The reactions of this module are summarized in Table 2. The kinetic rate constants are taken from the stochastic time constants found in [14]. This deterministic model can be tested by simulating it over a 100-second time course. Initially the cAMP level is set to 0 and the PKA level is set to 2500 molecules per cell. After 10 s the cAMP level is set to 270900 molecules per cell (equivalent to 0.015 mM). After 30 seconds the cAMP level is increased to 903000 (equivalent to 0.05 mM), and after 60 seconds the cAMP level is decreased to 270900 molecules per cell. For the cAMP level to affect the greatest control on the system, the difference in the level of free catalytic subunits of PKA between low and high cAMP levels should be as high as possible. Cyclic AMP activates PKA, therefore we expect to see an increased difference between active and inactive PKA when cAMP levels are physiologically high.

**Table 2:** Reactions of PKA Model A

**Reaction name**	**Formula**	**Rate law**
cAMP-PKA association	cAMP + PKA.x*cAMP ⇒ PKA.(x+1)*cAMP	*k*_*cAMPgain*_[PKA·x*cAMP][cAMP]

cAMP-PKA dissociation	PKA.x*cAMP ⇒ PKA.(x-1)*cAMP + cAMP	*k*_*cAMPloss*_[PKA·x*cAMP]

PKA dissociation	PKA*4cAMP ⇒ 2R*2cAMP + 2 C	*k*_*PKAdiss*_[PKA*4cAMP]

R-cAMP dissociation	R*2cAMP ⇒ R + 2 cAMP	*k*_*RcAMPdiss*_[R*2cAMP]

PKA association	2 R + 2 C ⇒ PKA	*k*_*PKAass*_[R]^2^[C]^2^

**Figure 2:**
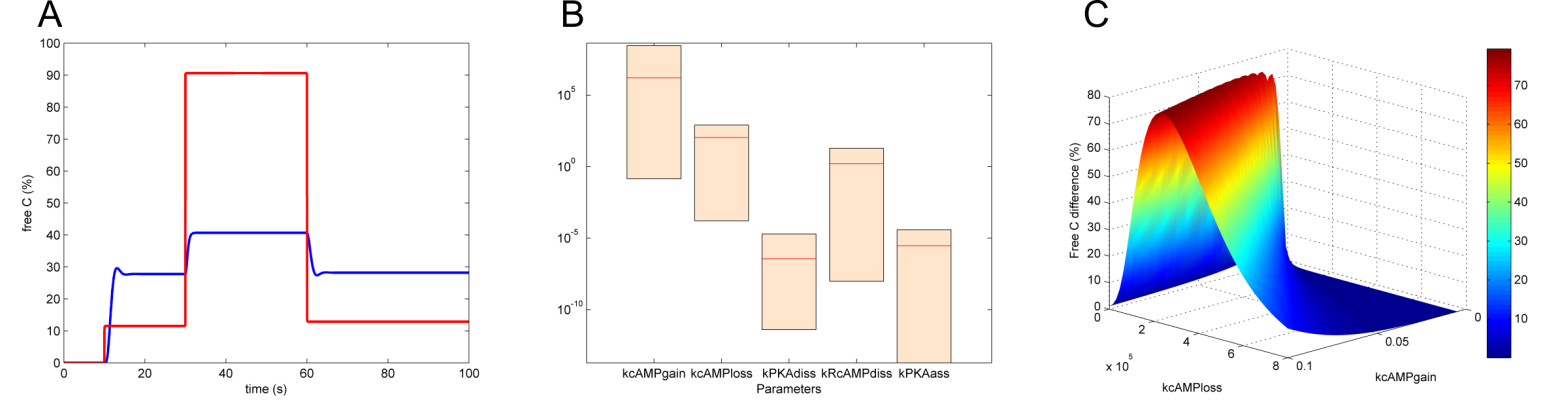
**Deterministic model of the PKA module**. (A) Simulation of PKA Model A (blue trace) and PKA Model B (red trace). The cAMP level is 0 initially, and is increased to 270900 molecules per cell (equivalent to 0.015 mM) after 10 seconds, increased to 909000 molecules per cell after 30 seconds, and decreased to 270900 molecules per cell after 60 seconds. (B) Steady state parameter sensitivity analysis carried out on the PKA module. (C) Parameter scan of PKA Model A. The greatest value for PKA difference (79.1%) is achieved when *k*_*cAMPgain *_= 0.1, *k*_*cAMPloss *_= 2.2 × 10^5^, *k*_*PKAdiss *_= 1 × 10^5^, *k*_*RcAMPdiss *_= 100, *k*_*PKAass *_= 1000.

As shown by the blue trace of Figure 2 (panel A) no free catalytic subunit is present when cAMP is set to zero. The model shows changes in the proportion of free catalytic subunits of PKA when cAMP is set to low (C_*low*_) and high (C_*high*_) levels. However the difference between the two states is not great – 27.7% when cAMP is low compared to 40.6% when cAMP is high. It is therefore important to optimize the model, and for this purpose parameter sensitivity analysis was carried out. As shown in Figure 2 (panel B), the parameter *k*cAMP*gain *is the most sensitive to variations in PKA level. The parameters of this model were scanned further to identify those which determined the highest difference between C_*low *_and C_*high*_. Figure 2 (panel C) shows how the difference between C_*low *_and C_*high *_depends on the parameters *k*cAMP*gain *and *k*cAMP*loss*. The peak values of this distribution were used to create an optimised model, named PKA Model B, whose simulation is shown by the red trace of Figure 2 (panel A). In PKA Model B, the level of C_*low *_now stands at ~10% whilst that of C_*high *_is approximately 90%.

With regards to modelling PKA activation, we wanted to test if the multi-reaction module used so far could be approximated with mass action kinetics or Michaelis-Menten type kinetics. We therefore generated two new PKA Models named C and D, respectively for mass action and Michaelis-Menten kinetics. In these new models, the reaction scheme becomes:



These models are defined by the following ODEs.

PKA Model C:

(6)

PKA Model D:

(7)

**Figure 3:**
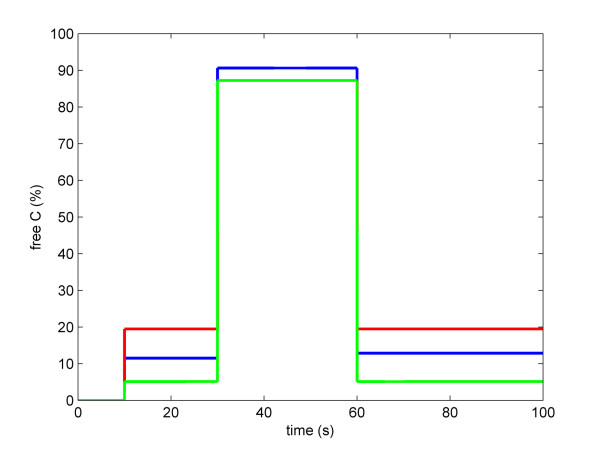
**Optimisation of the PKA model**. The blue trace shows the simulation of PKA Model B, the red trace – PKA Model C, and the green trace – PKA Model D. The cAMP level is 0 initially, and is increased to 270900 molecules per cell (equivalent to 0.015 mM) after 10 seconds, increased to 909000 molecules per cell after 30 seconds, and decreased to 270900 molecules per cell after 60 seconds.

We found that these simplified PKA modules could accurately approximate species levels of the optimized PKA Model B, with the following parameters: for PKA Model C, *kA *= 8.72e-17; *kR *= 1000; for PKA Model D, *V*_*max*_*f *= 1e-13; *K*_*M*_*F *= 1e7; *V*_*max*_*r *= 1000; *K*_*M*_*r *= 0.01 (Figure 3).

**Figure 4:**
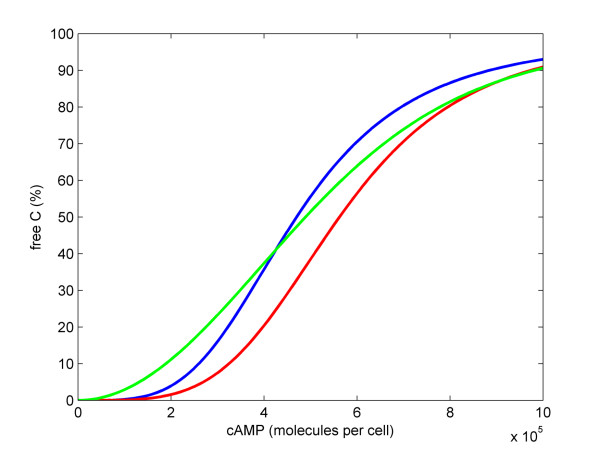
**Steady state levels of free C in the PKA models under various cAMP levels**. The blue trace shows the simulation of PKA Model B, the red trace – PKA Model C, and the green trace – PKA Model D. Parameters of PKA Model B are the same as in Figure 3. Parameters of PKA Model C are: *k*_*A *_= 8.72 × 10^-17^; *k*_*R *_= 1000. Parameters of PKA Model D are: *k*_*cat *_= 10^-13^; *K*_*mF *_= 10^7^; *V*_*maxR *_= 1000; *K*_*mR *_= 0.01.

We also compared steady state proportions of free catalytic subunit of PKA (*C*_*free*_) of each PKA model as a function of the cAMP concentration (Figure 4). At low cAMP concentrations, the Michaelis-Menten based model (PKA Model D) slightly over-estimated, while the mass action based model (PKA Model C) slightly underestimated the level of *C*_*free*_, respectively, in comparison to the optimised PKA Model B.

The results of simulating these models show that it is possible to simplify the PKA module greatly without loss of performance. It is preferable to use the mass action based module, as it has just three state variables and two parameters. This compares favourably to the complex PKA module which has nine state variables and four parameters. Therefore we adopted the mass action based module to construct the model of the entire cAMP pathway.

### Development and simulation of a conceptual model of the complete cAMP pathway

As a step towards developing a deterministic model of the complete cAMP pathway, we first constructed a conceptual model named Simplified cAMP Model A (Table 1). It consists of three ODEs (equations 8–10), based on mass action kinetics and uses unitless species concentrations and parameter values shown in Table 3. Equation 8 represents the combined G proteins activation and inactivation module, equation 9 – the PKA module, and equation 10 – cAMP synthesis and degradation.

**Table 3:** Parameters of Simplified cAMP Model A

**Parameter**	**Value**
GPR *k*_*F*_	0.1

GPR *k*_*R*_	0.01

PKA *k*_*F*_	0.1

PKA *k*_*R*_	0.1

*V*_*max *_AC	50

*K*_*m *_AC	1

*K*_*i *_AC	100

*V*_*max *_Pde1	10

*K*_*m *_Pde1	1

*V*_*max *_Pde2	1

*K*_*m *_Pde2	1

(8)

(9)

(10)

The conservation relationships in Simplified cAMP Model A are described below in equations 11–12 and represent the conservation of the total number of G proteins (GP), and that of total PKA molecules, respectively:

(11)

(12)

where GP_i _and GP_a _are the numbers of inactive and active G proteins, respectively. PKA_i _and PKA_a _are the number of inactive and active PKA molecules, respectively.

**Figure 5:**
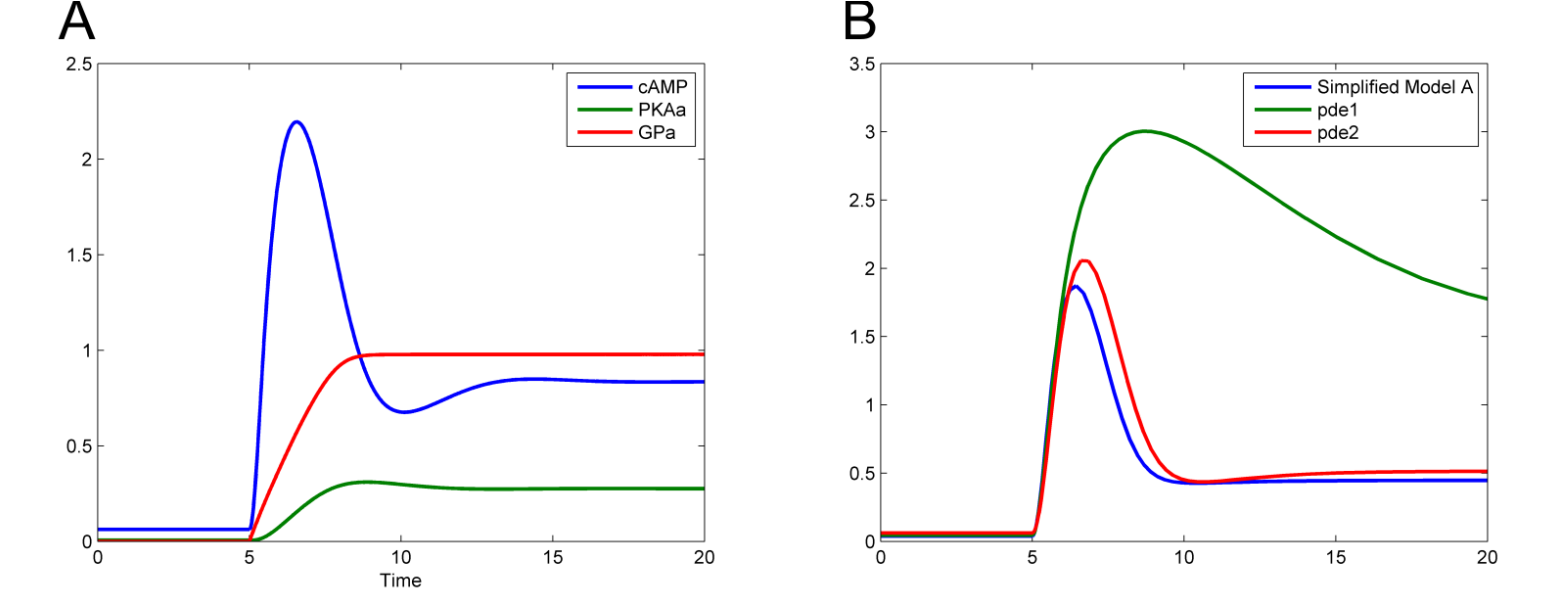
**Predictions of Simplified cAMP Model A**. (A) Species concentrations before and after a pulse of glucose. (B) Cyclic AMP levels of *pde1Δ *and *pde2Δ *mutants: blue trace – wild type; red trace – *pde2Δ*; green trace – *pde1Δ*. Glucose is increased to 5 after 5 seconds in both simulations.

The Simplified cAMP Model A was tested to see if it could reproduce the changes in cAMP levels observed experimentally during a glucose pulse. In a preliminary step, the initial concentrations of cAMP, PKA_i _and GP_i _were set to 1, and PKA_a_, GP_a _and glucose were set to zero. A steady state was then found, and subsequently all concentrations were set to their steady-state level. A simulation of the model was then run changing the glucose concentration to 5 after 5 time units. As shown in Figure 5 (panel A), a spike of cAMP was observed when the glucose concentration was increased and simultaneously GP and PKA activated. Apart from the slight dip in cAMP observed at time 10, the simulation accurately reproduces published experimental data [9,19,38].

To test if the model would also accurately reproduce phenotypic cAMP profiles of *pde1Δ *and *pde2Δ *mutants, the cAMP ODE (equation 10 defined above) was modified to remove the Pde1 and Pde2 reactions. The resultant "mutant" models were simulated as before, and as shown in Figure 5 (panel B), the simulations accurately reproduce the experimental data [19] (again with the exception of the slight dip in cAMP profile seen in the wild type and *pde2Δ *model mutants). We therefore conclude that this greatly simplified conceptual model is capable of reproducing the essential dynamics of changes in cAMP levels observed in response to glucose addition in wild-type as well as in the cAMP phosphodiesterase deletion mutants.

We then used this model to test the roles of the Krh proteins, which according to Harashima and Heitman [22] act by stabilizing the Ira proteins, whereas according to Peeters *et al. *[7,45] they directly inhibit PKA. Initially we incorporated the Krh proteins into the Simplified cAMP Model A based on their function proposed by Peeters *et al*. The model extended in this way is called Simplified cAMP Model B. The ODE for PKA has been modified accordingly to include the Krh proteins (equation 13). The rate of PKA activation is decreased by Krh, and the rate of PKA deactivation is increased by Krh:

(13)

The formation of the G protein complex was modeled with the following mass action equation:

(14)

We tested Simplified cAMP Model B to see if it could reproduce the results from studies on adenylate cyclase mutants by Peeters *et al. *[7]. For this purpose, adenylate cyclase was removed from the model. The adenylate cyclase deletion model (*cyr1Δ*) was simulated with cAMP concentration set to 1. The *GPA2*^*Q*300*L *^(Gpa2 constitutively active) mutant was modeled by setting the concentration of GP_a _to 1 and the parameter *V*_*maxGPdeact *_to 0. The *pde2Δ *mutant model was simulated as described earlier. The *krh *mutant was simulated fixing GP_a _levels to 1.

**Figure 6:**
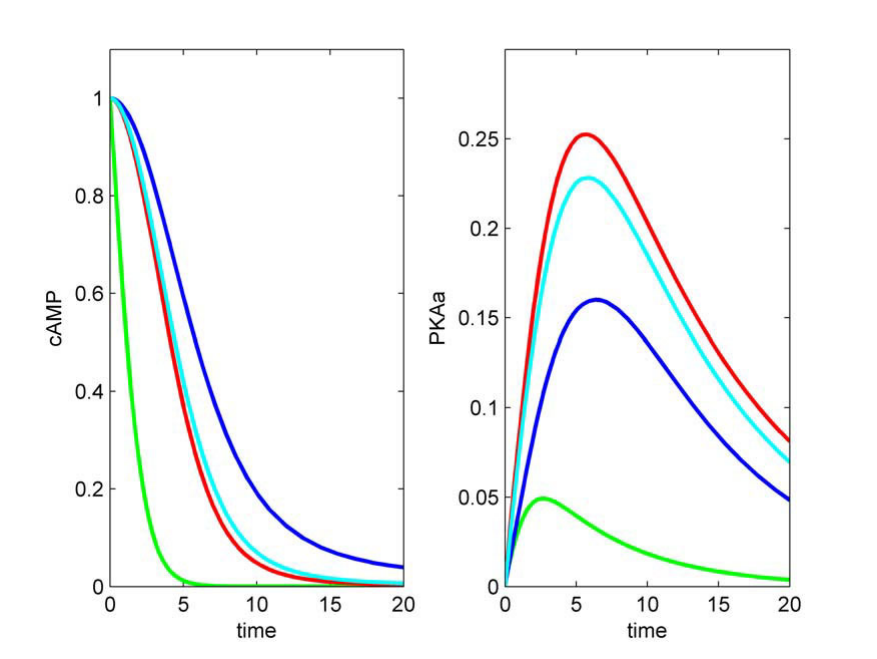
**Cyclic AMP and PKAa levels in Simplified cAMP Model B mutants when cAMP levels are set to 1 and PKAa set to 0**. Model mutant genotypes are: *cyr1Δ *(green), *cyr1Δpde2Δ *(blue), *cyr1Δpde2Δkrh1/2Δ *(red), *cyr1Δpde2Δ *GPA2^*Q*300*L *^(cyan).

As shown in Figure 6 the cAMP and PKA_a _levels of the mutant model simulations are in agreement with experimentally observed phenotypes. The *cyr1Δ *model mutant has near-zero steady state levels of cAMP and PKA, which corresponds well with the fact that a *cyr1Δ *mutant is nonviable. Deleting Pde2p in the model elevates cAMP and PKA_a _levels, a result which agrees with the observation that a *cyr1Δpde2Δ *mutant is viable if supplemented with external cAMP. Deletion of Krh in the model produces a further increase in PKA_a_, which is in agreement with the observation that these mutants require less exogenous cAMP for viability [45]. Simulation of the model gives results that correspond well to the observations of Peeters *et al.*, [7] when Krh is modeled as a direct inhibitor of PKA.

We attempted to make a model of Krh activity as proposed by Harashima and Heitman [22]. In the Simplified cAMP Model B, Krh is quickly reassociated with the G proteins, allowing the system to exert negative feedback. However, any feedback in the mechanism proposed by Harashima and Heitman [22] is impossible because the Ira proteins are degraded, and re-synthesis of these proteins could not be fast enough to allow the Ira proteins to inhibit the Ras proteins. Therefore in all further developments of the complete cAMP pathway models Krh was retained as a direct inhibitor of PKA.

### Modelling the complete cAMP pathway's response to glucose

**Figure 7:**
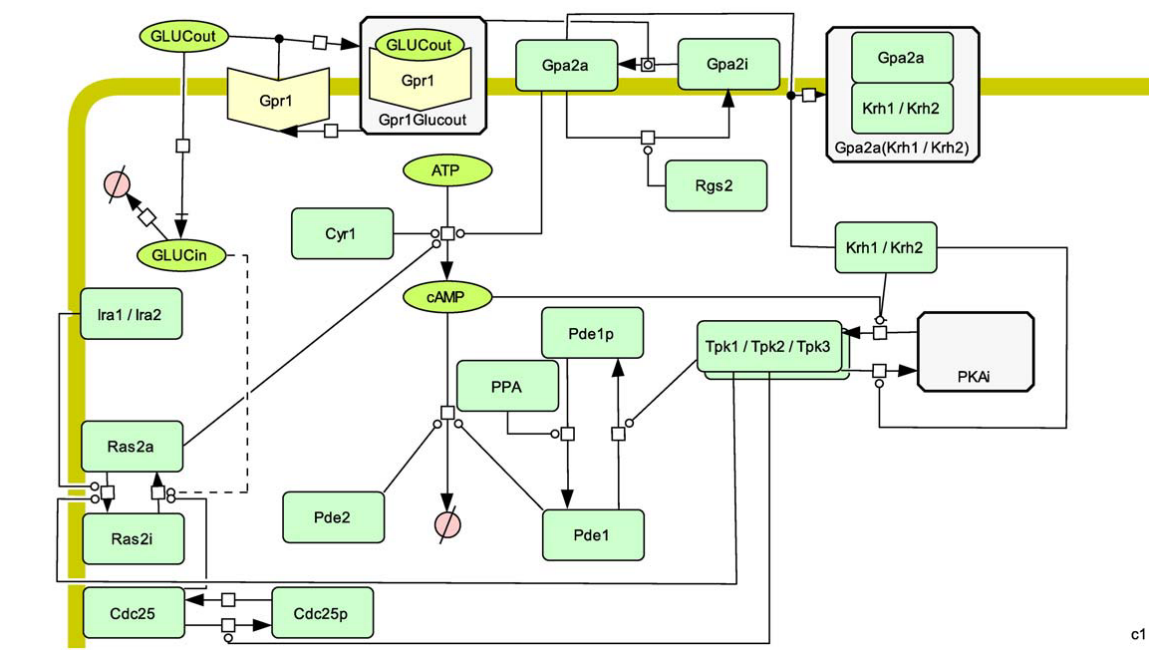
**Schematic representation of the Complete cAMP model, using the SBGN notation**.

The simplified conceptual model allowed us to capture the essential dynamics of the cAMP pathway and also to successfully incorporate the role of the Krh proteins. In order to fully understand the dynamics of this complex pathway we created a deterministic model which includes all components of the cAMP pathway and their interrelationships as currently reported. This Complete cAMP Model (Table 1 and Figure 7) consists of several distinct "modules" which are described below.

### Glucose import and metabolism

The import of glucose was modelled using the following equation, as in [46]:

(15)

where *v*_*tr *_is the rate of transport (in mM per second), *s *is the extracellular glucose concentration, *p *is the intracellular glucose concentration, *K*_*M *_is the Michaelis constant (in mM) and *K*_*i *_is the interaction constant.

The metabolism of glucose via glycolysis was summarized with mass action kinetics, so that the intracellular glucose concentration did not exceed 1.5 mM during simulation, as described [46].

### Gpa2 and Krh

As described earlier, Gpa2 is activated by Gpr1, and Gpr1 is activated by extracellular glucose. The activation of Gpr1 is modelled with mass action kinetics, whereby Gpr1 forms a complex with extracellular glucose. The activation of Gpa2 is based on mass action kinetics, with activated Gpr1 as an essential activator. Deactivation of Gpa2 is modelled using a basal rate of deactivation (representing the intrinsic GTPase activity of Gpa2), which can be enhanced by Rgs2. The binding of Gpa2 to Krh to form a complex is represented with simple mass action kinetics.

### Ras2

Ras2 is very challenging to model because a large number of molecular species are involved in its regulation. It is directly activated by Cdc25, but it is activated indirectly by glucose. We chose to model the activation of Ras2 using general hyperbolic modifier kinetics. In this reaction, glucose acts as a modifier which increases the rate of the reaction, but the reaction is dependent on Cdc25. The deactivation of Ras2 was modelled using modified mass action kinetics with Ira as an activator. This captured the intrinsic GTPase activity of Ras2.

### Adenylate Cyclase

Adenylate cyclase is represented as a Michaelis-Menten enzyme, with the following modifications. Activated Gpa2 and activated Ras increase the *k*_*cat *_of adenylate cyclase, increasing the maximum activity of the enzyme. In order to simplify the model, the substrate for adenylate cyclase (ATP) is not included, as the intracellular concentration of ATP is always greatly in excess of the cAMP concentration.

### PKA

PKA is modelled using the mass action kinetics module with the addition of the actions of the Krh proteins described earlier. The forward reaction (PKA dissociation) is inhibited by Krh, and the backward reaction (PKA association) is activated by Krh.

### The Phosphodiesterases

Pde2 is represented as a Michaelis Menten enzyme with a *K*_*m *_value of 0.002 mM, determined by parameter estimation (Table 4). Pde1 has been shown to be activated by phosphorylation, so the phosphorylated form has a lower *K*_*m *_and higher *k*_*cat *_than the dephosphorylated form. For this reason, Pde1 is represented by two species – the phosphorylated and the dephosphorylated form of Pde1p, respectively.

**Table 4:** Parameters of the complete cAMP pathway Model

**Parameter name**	**Value**	**Units**	**Source**
Glucose transport *K*_*M*_	0.08	mM	[37]

Glucose transport *V*	1.7	mmol/s	[37]

Glucose transport *K*_*i*_	0.91		[37]

Glucose Utilisation *k*_*F*_	0.03	l/s	This work

Gpr1 Glucose association *k*_1_	0.003	l^2^/mmol*s	This work

Gpr1 Glucose dissociation *k*_1_	0.14	l/s	This work

Gpa2 activation *k*_*A*_	57682.6	l^2^/mmol*s	This work

Gpa2 deactivation *k*_*F*_	0.899	l/s	This work

Gpa2 deactivation *k*_*A*_	12989.4	l^2^/mmol*s	This work

Gpa2-Krh association *k*_*F*_	391089.6	l^2^/mmol*s	This work

Gpa2-Krh dissociation *k*_*F*_	6.12	l/s	This work

Ras2 activation *k*_*cat*_	0.74	l/s	This work

Ras2 activation *K*_*M*_	1.38 × 10^-3^	mmol/l	This work

Ras2 activation *K*_*d*_	0.044	mmol/l	This work

Ras2 activation *a*	32.9		This work

Ras2 activation *b*	63.8		This work

Ras2 deactivation *k*_*F*_	0.042	l/s	This work

Ras2 deactivation *k*_*A*_	519.8	l^2^/mmol*s	This work

cAMP synthesis *k*_*cat*_*Gpa2*	2933.9		This work

cAMP synthesis *k*_*cat*_*Ras2*	650		This work

cAMP synthesis *K*_*M*_	4 × 10^-3^	mM	This work

PKA activation *k*_*F*_	7.6 × 10^8^		This work

PKA activation *k*_*I*_	100	l/mmol	This work

PKA deactivation *k*_*F*_	19.9		This work

PKA deactivation *k*_*A*_	2.2 × 10^4^	l/mmol	This work

Cdc25 phosphorylation *k*_*cat*_	0.18	l/s	This work

Cdc25 phosphorylation *K*_*M*_	5.2 × 10^-3^	mmol/l	This work

Cdc25 dephosphorylation *k*_*cat*_	2.52	l/s	This work

Cdc25 dephosphorylation *K*_*M*_	1.6 × 10^-2^	mmol/l	This work

Pde1 phosphorylation *k*_*cat*_	6.82	l/s	This work

Pde1 phosphorylation *K*_*M*_	8.6 × 10^-3^	mmol/l	This work

Pde1 dephosphorylation *k*_*cat*_	2.4	l/s	This work

Pde1 dephosphorylation *K*_*M*_	1.07 × 10^-3^	mmol/l	This work

cAMP hydrolysis (Pde1) *k*_*cat*_	1.1	l/s	This work

cAMP hydrolysis (Pde1) *K*_*M*_	0.85	mmol/l	This work

cAMP hydrolysis (Pde1p) *k*_*cat*_	25.25	l/s	This work

cAMP hydrolysis (Pde1p)*K*_*M*_	6 × 10^-7^	mmol/l	This work

cAMP hydrolysis (Pde2)*k*_*cat*_	1	l/s	This work

cAMP hydrolysis (Pde2) *K*_*M*_	0.002	mmol/l	[15]

**Figure 8:**
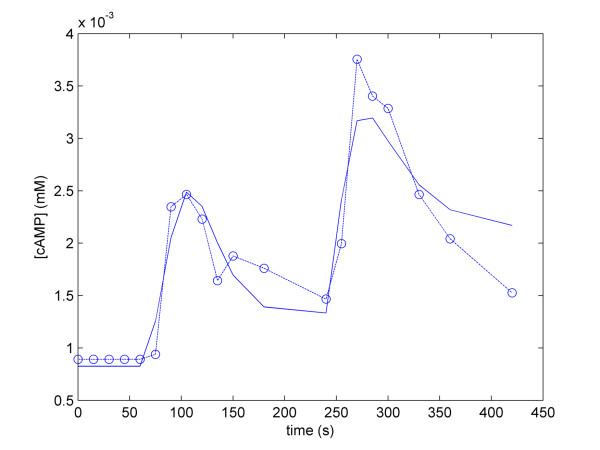
**Result of parameter estimation of the Complete cAMP Model**. The blue dotted trace represents cAMP data from [35], and the solid blue trace represents cAMP levels computed by the model.

The model was written in SBML format [36] and is included as Additional file 1. The pathway diagram was constructed using CellDesigner [47], incorporating the Systems Biology Graphical Notation (SBGN) scheme . The representation of the model is shown in Figure 7, and its reactions and rate laws are shown in Table 5. The parameters of the Complete cAMP Model are given in Table 4, including both estimated and experimentally derived parameters. The cAMP data used for the parameter estimation were taken from [38], where 5 mM glucose was added to glucose-starved cell suspension after 60 seconds, followed by the addition of 100 mM glucose after 240 seconds. The cAMP profile (Figure 8) computed by simulation of our Complete cAMP Model after parameter estimation is in good agreement with previous observations.

**Table 5:** Reactions of the Complete cAMP pathway Model

**Reaction name**	**Substrates**	**Products**	**Rate law**
Glucose transport (reversible)	Gluc_out_	Gluc_in_	

Glucose metabolism	Gluc_in_		*k*_*f *_[Gluc_in_]

Gpr1-Glucose Association	Gpr1, Gluc_out_	Gpr1Glucout	*k*_*f *_[Gpr1] [Gluc_out_]

Gpr1-Glucose dissociation	Gpr1Gluc_out_	Gpr1,Gluc_out_	*k*_*f *_[Gpr1Gluc_out_]

Gpa2 activation	Gpa2i	Gpa2a	*k*_*A *_[Gluc_out_] [Gpa2i]

Gpa2 deactivation	Gpa2a	Gpa2i	(*k*_*A *_[Rgs2] + *k*_*f*_) [Gpa2a]

Gpa2-Krh association	Gpa2a,Krh	Gpa2aKrh	*k*_*f *_[Gpa2a] [Krh]

Gpa2-Krh dissociation	Gpa2aKrh	Gpa2a,Krh	*k*_*f *_[Gpa2aKrh]

Ras2 activation	Ras2i	Ras2a	

Ras2 deactivation	Ras2a	Ras2i	(*k*_*A*_[Ira]+*k*_*f*_)·[Ras2a]

cAMP synthesis		cAMP	

PKA activation	PKAi	2*C	

PKA deactivation	2*C	PKAi	*k*_*f*_[C]^2^·(1 + (*k*_*A*_[Krh]))

Cdc25 phosphorylation	Cdc25	Cdc25P	

Cdc25 dephosphorylation	Cdc25P	Cdc25	

Pde1 phosphorylation	Pde1	Pde1P	

Pde1 dephosphorylation	Pde1P	Pde1	

cAMP hydrolysis (Pde1)	cAMP		

cAMP hydrolysis (Pde1P)	cAMP		

cAMP hydrolysis (Pde2)	cAMP		

**Figure 9:**
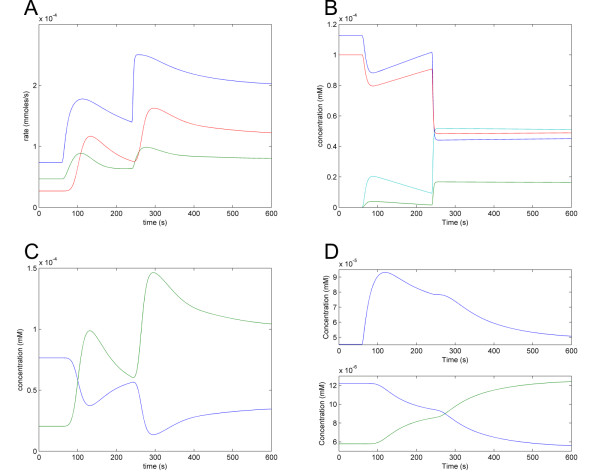
**Predictions of the Complete cAMP Model**. (A) cAMP synthesis and hydrolysis rates. Glucose is increased to 5 mM at time 60, and increased to 100 mM at time 240. Blue trace: rate of cAMP synthesis. Red trace: rate of cAMP hydrolysis by Pde1. Green trace: rate of cAMP hydrolysis by Pde2. (B) Levels of species in the Gpa2 module. Blue trace: inactive Gpa2. Green trace: active Gpa2. Red trace: Krh. Cyan trace: complex of activated Gpa2 and Krh. (C) Levels of active (blue trace) and inactive (green trace) PKA. (D) Levels of Ras2a and Cdc25 in response to 5 mM glucose at time 60sec, and 100 mM at 240 sec. Top part: Ras2a (blue trace); bottom part: phosphorylated (green trace) and unphosphorylated (blue trace) Cdc25.

The Complete cAMP Model illustrates several important features of the pathway. The balance of flux between cAMP synthesis and hydrolysis (Figure 9, panel A) demonstrates that Pde1p is more important than is Pde2p for controlling the cAMP levels following glucose pulses, as the effect of Pde1p on the rate of cAMP hydrolysis is much greater than that of Pde2p. Furthermore, the level of active Gpa2 is proportional to the level of extracellular glucose, and the level of Krh drops as it forms a complex with activated Gpa2 (Figure 9, panel B). Importantly, the proportion of active PKA (Figure 9, panel C) is not directly proportional to the cAMP level (Figure 8), allowing PKA to exert negative feedback on the cAMP level, even when the cAMP level drops. PKA exerts this feedback by activating Pde1p (Figure 9, panel A) and deactivating Ras2 via phosphorylation of Cdc25 (Figure 9, panel D). The latter mechanism is a feature of the Complete cAMP Model but not of the Simplified cAMP Model A and explains why cAMP level can come down after a glucose pulse in the Complete cAMP Model but not in the Simplified cAMP Model A.

## Discussion

We have successfully created a series of deterministic mathematical models to investigate the cAMP pathway in *S. cerevisiae*. These range from simplified, conceptual models of the pathway, to an extensive model that fits experimental data. We were able to build a simplified model of the PKA module, containing only two variables and two parameters, without compromising the behaviour of the system. The simplification of the PKA module demonstrates the power of deterministic models. The components of this pathway are present in high abundance (proteins in thousands, nucleotides in millions per cell), making a deterministic model better suited than a stochastic one (we note also that we are not seeking to model potentially hundreds of kinds of protein molecule with different post-translational modifications).

In our PKA Model, the activation of PKA is worthy of particular attention. In previously published models, PKA activity was directly proportional to the cAMP level [14]. However, it has been proposed that PKA autophosphorylation provides a feed-forward mechanism for PKA activation [48], as Tpk1p is phosphorylated following a glucose pulse [21]. Alternatively, it is proposed that Krh inhibits PKA, and this inhibition is removed when Krh is recruited to activated Gpa2 [45]. Our Simplified cAMP Model B shows that the latter scenario is more likely, as this model corresponds well with observable phenotypes.

Our Simplified cAMP Model shows that the basic dynamics of the pathway in response to glucose can be explained with a relatively straightforward feedback mechanism. The activation of PKA by cAMP, followed by the activation of Pde1 and the inhibition of adenylate cyclase is sufficient to produce a characteristic "spike" of cAMP, followed by the emergence of a new steady state level of cAMP and PKA. This model has been tested by creating phosphodiesterase deletion mutant models (Figure 5, panel B). Deleting Pde2 in the model results in a higher steady state level of cAMP, but it does not significantly affect the cAMP spike. This phenotype is indeed found in yeast *pde2Δ *mutants [3]. However, removing Pde1 from the model results in a cAMP spike with increased peak height and duration, which is comparable to that experimentally determined in *pde1Δ *mutant [19].

In the Simplified cAMP model A, a slight dip in the level of cAMP can be seen before the cAMP level reaches a steady state after a pulse of glucose. Although this slight oscillation is not widely noted in the literature, it is possible to observe it in some experiments [38]. The presence of the slight oscillation in the model is dependent on the parameters of the model and the glucose concentration. It remains to be seen whether this oscillation is truly present in all or any circumstances.

The Simplified cAMP Model B (which incorporates the Krh proteins) demonstrates the significance of the negative feedback. Furthermore, it shows that this feedback is possible if the Krh proteins were acting as direct inhibitors of PKA as proposed by Peeters *et al*. [7,45] rather than stabilising the Ira proteins as proposed by Harashima and Heitman [22]. At the same time, it predicts that cAMP levels should decrease more rapidly in the *cyr1Δpde2Δkrh1Δkrh2Δ *mutant than in the *cyr1Δpde2Δ *mutant. It will be interesting to see if these mutants behave in the way predicted by our models.

Although the Simplified cAMP Model could account for the majority of the behaviour of the cAMP pathway, there were exceptions. Most notably, in simulations of the *pde1Δ *mutant model, the steady state level of cAMP became significantly higher after a glucose pulse than it was before (Figure 5, panel B). This is not seen experimentally, where there is little difference between the post-glucose cAMP levels seen in a wild type and *pde1Δ *mutant [19]. This feature of the Simplified cAMP Model prompted us to develop the Complete Model. Our Complete cAMP Model represents the first effort to consolidate all the known elements of the cAMP pathway into one deterministic mathematical model. In addition to this, we have fitted the parameters of our model to experimental data. The fact that the complete cAMP pathway model can reproduce cAMP levels found in the literature indicates that the model is a reliable *in silico *approximation of the *in vivo *system. Furthermore, our model has other advantages. Firstly, as a deterministic model, it is computationally inexpensive to simulate and easy to analyze. Secondly, it represents a physiologically realistic steady state before glucose is introduced, in that the cAMP level is not zero. This contrasts with the model found in [14], in which the cAMP level is set to zero before glucose addition, which is biologically impossible, as cAMP is required for cell viability. After glucose addition, the model correctly represents the dynamical changes in cAMP level, until the cAMP level reaches a new steady state.

The models of the cAMP pathway described in this study make a number of predictions that could be tested experimentally. As a matter of further investigations in our lab, different species would be characterized following a pulse of glucose in terms of phosphorylation (Pde1p, Cdc25p), GTP loading (for Gpa2p), changes in cAMP levels (in *cyr1Δpde2Δkrh1Δkrh2Δ *in comparison to *cyr1Δpde2Δ*). Our Complete cAMP Model will no doubt be improved and tested further in the future. As more parameters are derived through experimentation, they can be included into the model to replace currently estimated parameters. We provide this model in SBML (Additional file 1), so that it can be easily expanded as scientific knowledge increases. For example, details on the mechanism of glucose activation of Ras2 could be incorporated when this mechanism is elucidated.

This model could be integrated with models of other pathways, a good example being that of the cell cycle, given the fact that cell cycle progression is controlled partly by the cAMP pathway [49]. It could also be integrated with a metabolic model such as the community consensus version recently published [50] via known PKA targets. Furthermore it could be adapted to other organisms such as the human fungal pathogen *Candida albicans*, as it is well documented that the cAMP pathway plays a key role in regulating virulence [51].

## Conclusion

We report a deterministic mathematical model of the cAMP-mediated signal transduction pathway in *S. cerevisiae*. The model is easier to compute and simulate as it has a reduced number of variables and parameters in comparison to previously reported stochastic model of this pathway. Furthermore, our model contains components such as the regulatory Krh proteins that have not been included before. It is able to simulate accurately experimentally derived patterns of cAMP changes observed in different pathway mutants in response to glucose addition. We suggest that it is suitable for integration with other models such as that of the cell cycle or metabolism and that it could be adapted to medically important yeast species such as the human fungal opportunistic pathogen *C. albicans*.

## Authors' contributions

TW carried out the modeling work and drafted the manuscript. J-MS and DK participated in different stages of the modeling work and in writing of the manuscript. LS conceived of the study and participated in its design and coordination and helped to draft the manuscript. All authors read and approved the final manuscript.

## Supplementary Material

Additional file 1 Complete cAMP Model in SBML format. Click here for file
